# Disparities in treatment modalities and survival among older patients with high-grade serous ovarian cancer

**DOI:** 10.1186/s12905-024-02938-y

**Published:** 2024-02-07

**Authors:** Yan Cai, Tong Shu, Hong Zheng

**Affiliations:** Key laboratory of Carcinogenesis and Translational Research (Ministry of Education/Beijing), Department of Gynecologic Oncology, Peking University Cancer Hospital & Institute, Beijing, 100142 China

**Keywords:** High-grade serous ovarian cancer, Older patients, Chemotherapy, Surgery, Treatment disparities

## Abstract

**Background:**

Undertreatment of ovarian cancer is common among older women. We aimed to evaluate the treatment modalities offered to older patients and their impact on overall survival (OS).

**Methods:**

The study identified 5,055 patients with high-grade serous ovarian cancer and 3584 patients with advanced stage (IIIC + IV) disease from the Surveillance, Epidemiology, and End Results (SEER) database from January 1, 2010, to December 31, 2017. We performed comparisons of OS and ovarian cancer-specific survival (OCSS) across age groups using a Cox proportional hazards model.

**Results:**

Very elderly patients (≥ 75 years old) received treatment with significantly less surgical complexity, such as no lymphadenectomy (59.7% vs. 48.6%; *p* < 0.001) and a lower rate of optimal debulking surgery (44.0% vs. 52.7%; *p* < 0.001), as well as lower rates of chemotherapy (78.2% vs. 89.4%; *P*<0.001) and standard treatment (70.6% vs. 85%; *p* < 0.001). High proportions of both very elderly and elderly patients received neoadjuvant chemotherapy (NACT), with no significant difference (38.7% vs. 36.2%; *P* = 0.212). Patients aged ≥ 75 years had significantly worse OS and OCSS.

**Conclusion:**

With increasing age, the survival rate of women with ovarian cancer decreases significantly. Noticeably fewer ovarian cancer patients aged over 75 years receive standard treatments, and more very elderly patients are treated with NACT.

**Supplementary Information:**

The online version contains supplementary material available at 10.1186/s12905-024-02938-y.

## Introduction

Among women in the United States, ovarian cancer is the fifth most deadly malignancy, and predictions based on the national statistics suggest that there will be 19,710 new cases and 13,270 deaths in 2023 [1]. Generally, ovarian cancer is diagnosed in older women. Specifically, over half of all new cases are diagnosed in women over 65 years of age, and approximately 60% of patients are over the age of 65 [2]. Due to a lack of effective screening strategies and the lack of early symptoms, more than 50% of patients are diagnosed with advanced ovarian cancer [1]. Compared to younger patients, older women often present with advanced disease stages and have poorer outcomes. More than 65% of women over 75 years old while 39% of women under 50 years old have distant cancer. Additionally, the 5-year survival rate among women over 50 is 16% and that among women under 50 is 43% [3]. Most patients undergo comprehensive staging surgery, primary debulking surgery (PDS) or neoadjuvant chemotherapy (NACT) and interval debulking surgery (IDS), followed by adjuvant chemotherapy [4]. During PDS or IDS, complete gross resection (CGR) or a residual disease extent ≤ 1 cm is optimal for improving survival but may be accompanied by increased surgical morbidity and require more extensive surgery [5]. As older patients are frailer and more prone to complications, this type of treatment remains controversial [6]. Physiological changes associated with aging, such as kidney and liver function declines, can affect drug metabolism and chemotherapy effectiveness, increasing the risks of toxicities and other adverse effects [7]. Therefore, along with psychological and practical factors, including inherent age bias, older women with ovarian cancer receive less intensive treatment and experience earlier treatment discontinuation than younger women [8]. In clinical trials, older patients with ovarian cancer have historically low enrollment rates, which leaves a lack of data regarding their management [9]. However, as life expectancy increases, as the older population ages, ovarian cancer incidence is expected to rise. Consequently, oncologic management in this population is a critical issue that needs to be addressed.

The aims of this study were to evaluate the management of elderly (aged 65–74 years) and very elderly patients (aged ≥ 75 years) with high-grade serous epithelial ovarian cancer (EOC), which is the histological subtype of over 70% of all ovarian cancers [10], and to investigate treatment patterns and survival disparities by age in the updated SEER database.

## Methods

### Data sources and variables

This retrospective population-based study used the Surveillance, Epidemiology, and End Results (SEER) database. The data of the target patients were extracted with SEER*Stat Version 8.4.0.1 (SEER ID: 11,296-Nov2021). Based on the November 2021 submission, the April 2022 report contained population-based data from 17 cancer registries covering approximately 28% of the US population from 1975 to 2019 and provided complete information about patient demographics, tumor characteristics, diagnosis, treatment, and follow-up. Since the SEER database released public data, the present study did not require informed patient consent and was exempt from review by the Beijing Cancer Hospital ethics committee.

In the SEER database, data on sociodemographic and tumor characteristics were collected. Patients were divided into two cohorts: elderly women (aged 65–74 years) and very elderly women (aged ≥ 75 years). Marital status was classified as one of three categories: married, single, or unknown. In the SEER dataset, the 7th edition AJCC staging results for OC were converted to the 2014 staging of the International Federation of Gynecology and Obstetrics (FIGO). Tumor size was divided into the following five categories based on maximum diameter: ≤50 mm, 51–100 mm, 101–200 mm, > 200 mm and unknown. According to the number of LNs removed, we classified lymphadenectomy extent into four groups: 0 LNs (non-LND), 1–9 LNs, 10–19 LNs, and ≥ 20 LNs. The type of residual disease was classified as optimal or suboptimal; optimal was defined as no or microscopic residual disease (0–1 cm), while suboptimal was defined as macroscopic residual disease (> 1 cm). The SEER database provides information on whether chemotherapy was definitely performed; however, detailed information on regimen and cycle was unavailable.

The study outcomes were OS (overall survival) and OCSS (ovarian cancer specific survival) rates calculated from date of death, and date of cancer-related death. The survival time was measured in months, and OS was calculated from the date of diagnosis until death from all causes or until the last follow-up in November 2019. OCSS was calculated as the time from the date of diagnosis to cancer-related death.

### Cohort selection

We selected patients with ovarian serous cancer between 2010 and 2017 using the ICD-O-3 primary site code C56.9 (ovary) and morphology codes 8140/3, 8380/3, 8441/3, 8460/3, and 8461/3. As recommended by the 2014 WHO guidelines, we reclassified high-grade endometrioid tumors (grades 3–4) as high-grade serous carcinomas [11]. Patients with no confirmed diagnosis by histology, a primary tumor other than ovarian serous carcinoma, a survival time less than one month or of unknown duration, aged less than 65 years, or with well-differentiated (G1 and G2) disease were excluded from the study cohort (Online Supplemental Table 1).

### Statistical analysis

Comparisons of clinical and demographic characteristics were performed using the chi-squared test. Independent predictors of OS were identified by Cox regression. All data were analyzed using IBM SPSS Statistics for Windows/Macintosh, version 20 (IBM Corp, Armonk, NY, USA). Kaplan-Meier curves and log-rank tests were used to calculate OS rates. Kaplan–Meier survival curves were plotted using GraphPad Prism 7.0.0 (GraphPad Software, San Diego, CA, USA), and *P* values < 0.05 were considered statistically significant.

## Results

### Baseline demographic and clinicopathological characteristics

Between 2010 and 2017, 5055 patients met the inclusion criteria. The demographic and clinical characteristics of the whole cohort according to age group are reported in Table 1. The distribution of the two age groups was as follows: 3190 women (63.1%) were aged 65–74 years, and 1865 women (36.9%) were aged 75 years or older. The majority of the patients were white, married and in an advanced FIGO stage (III + IV): 82.8% in the 65–74-year group and 81.4% in the ≥ 75-year group. There were significant differences concerning the presence of lymph node metastasis, with 1583 (49.6%) of the elderly patients having undergone lymphadenectomy and 21.7% of the lymph nodes (LNs) being positive; however, only 714 (38.3%) of the very elderly patients underwent the same procedure, with 17.9% of the LNs being positive. Chemotherapy was administered to 89.4% (2851/3190) of the elderly patients and 78.2% (1458/1865) of the very elderly patients (*P* < 0.001).

### Treatment characteristics of women with advanced-stage disease

The treatment characteristics of the 3584 patients with an advanced FIGO stage (IIIc and IV) are presented according to age group in Table 2. There were 2296 women (64.1%) aged 65–74 years and 1288 women (35.9%) aged 75 years or older. There was no significant difference between the two cohorts regarding FIGO stage at diagnosis. The very elderly patients had significantly lower rates of pelvic and para-aortic lymphadenectomies (38.1% for ≥ 75 years, 45.6% for 65–74 years) with fewer LNs removed among those who underwent lymphadenectomy (*p* < 0.001). A total of 2860 (79.8%) patients received standard treatment for advanced ovarian cancer, defined as both chemotherapy and surgery, but fewer very elderly patients received the standard procedures (70.6% vs. 85% for the elderly patients, *p* < 0.001). There was a higher rate of optimal debulking surgery in the elderly group (52.7% among those aged 65–74 years and 44.0% among those aged ≥ 75 years, *p* < 0.001).

### Multivariate survival analysis

We performed multivariate Cox regression among all of the included patients to identify prognostic factors. Age, marital status, race, laterality, stage, Ca-125 level, tumor size, lymphadenectomy status, and chemotherapy were all found to be related to overall survival (OS). Notably, increased age showed unfavorable effects on OS, hazard ratio (HR) for the very elderly group of 1.403 with the elderly group as the reference (Online Supplemental Fig. 1). We found a similar trend when we performed multivariate Cox regression among those with advanced disease (HR was 1.287). Stage IV disease, no lymphadenectomy, no surgery, neoadjuvant chemotherapy, no standard treatment and suboptimal cytoreductive surgery were associated with a worse OS (Online Supplemental Fig. 2).

### Survival results

**Fig. 1:**
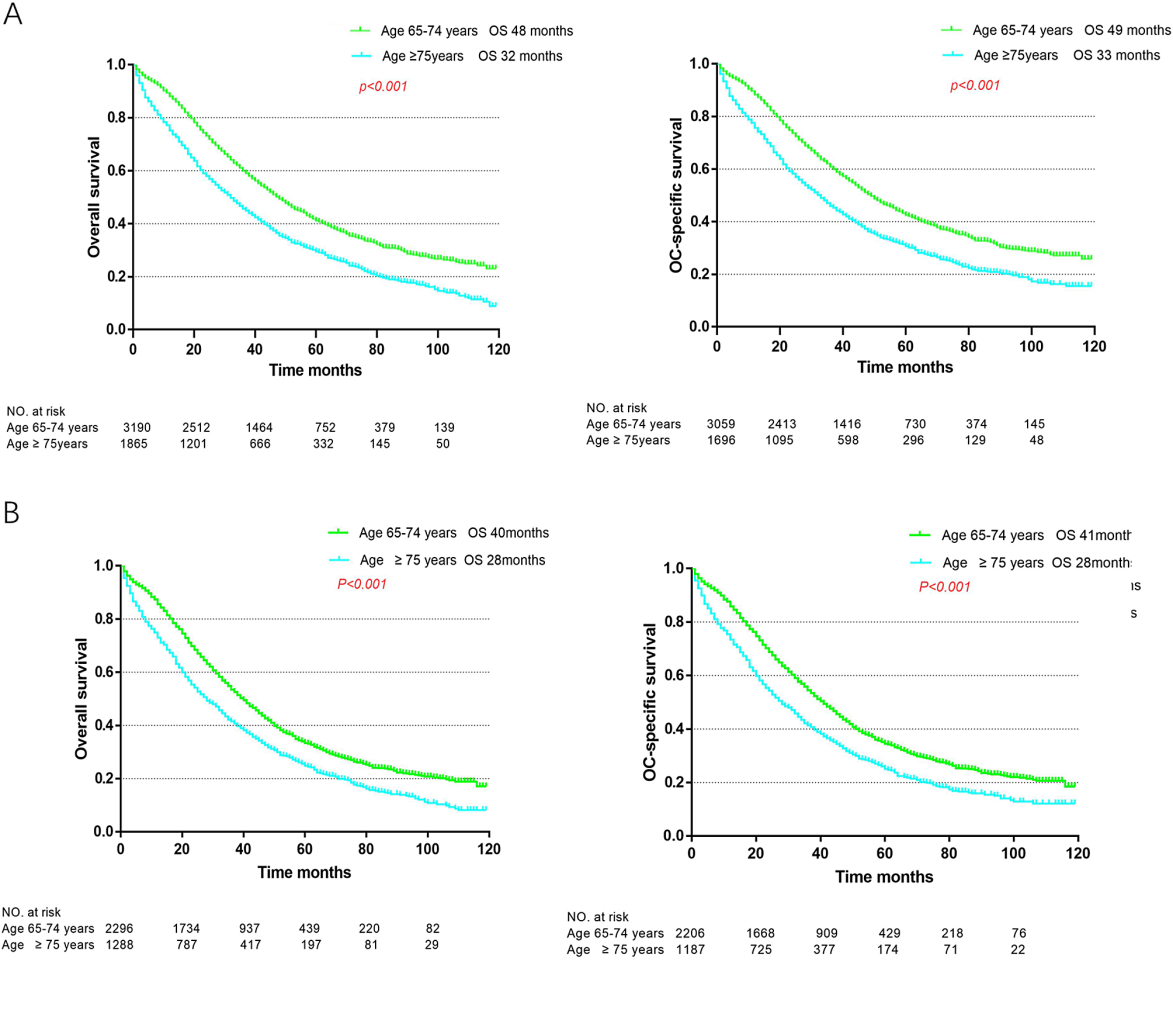
Overall survival and ovarian cancer (OC) specific survival by age group of all patients (**A**); Overall survival and ovarian cancer (OC) specific survival by age group of advanced stage patients (**B**). The number at risk in the weighted dataset is shown

The median follow-up time for OS was 44.4 months (range: 1–119 months) in all patients and 40.5 months (range: 1–119 months) in advanced-stage patients. The median OS was 32 months for the very elderly cohort and 48 months for the elderly cohort, and the ovarian cancer-specific survival (OCSS) durations were 33 and 49 months, respectively (Fig. 1A). Among the advanced-stage patients, the median OS durations for the very elderly and elderly cohorts were 28 and 40 months, and the OCSS durations were 28 and 41 months, respectively (Fig. 1B).

**Fig. 2:**
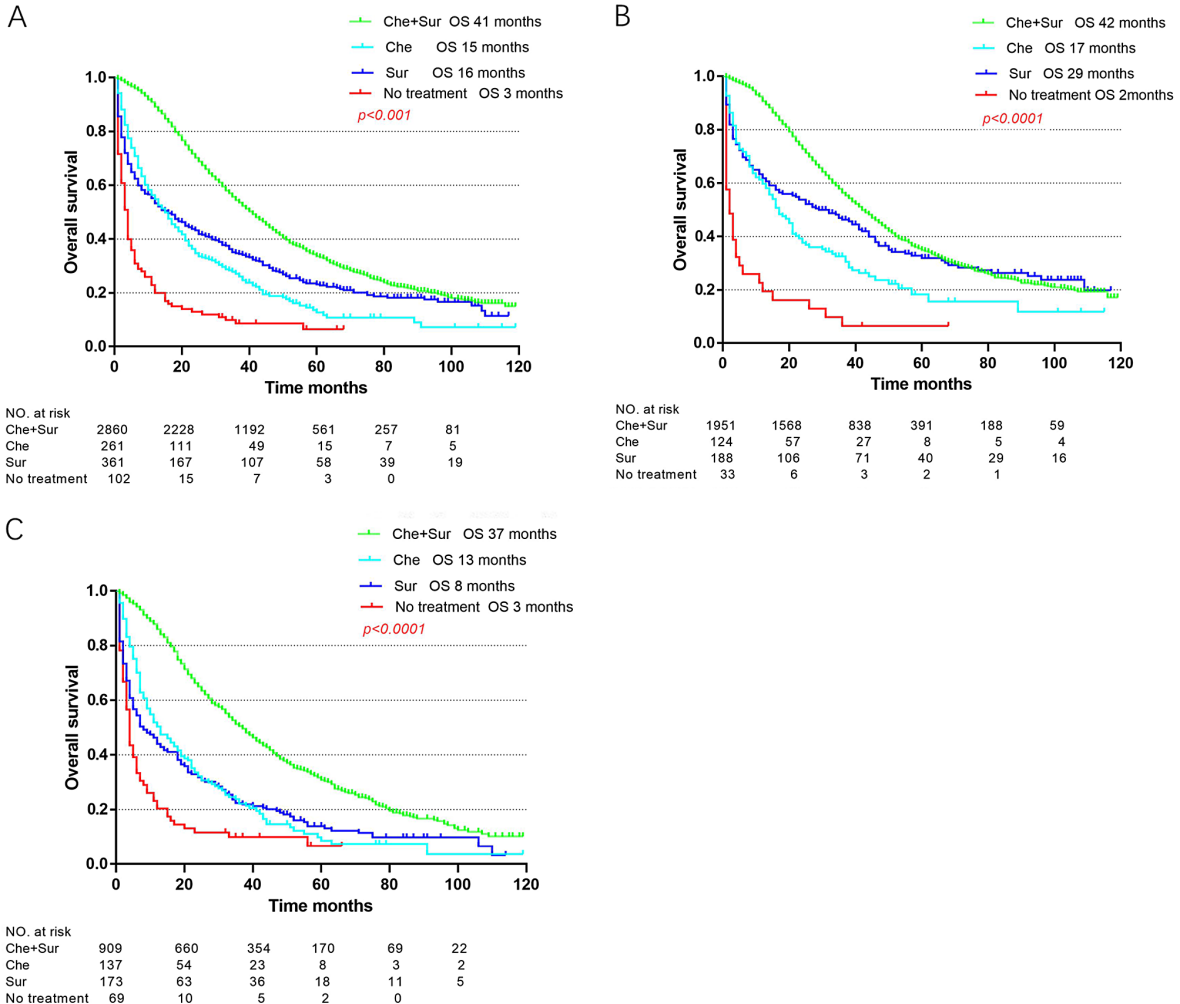
Stratified survival analyses by treatment group. (**A**) Overall survival of all advanced stage patients. (**B**) Overall survival of the elderly group. (**C**) Overall survival of the very elderly group. Abbreviations: Che + Sur, chemotherapy + surgery; Che, chemotherapy; Sur, surgery

**Fig. 3:**
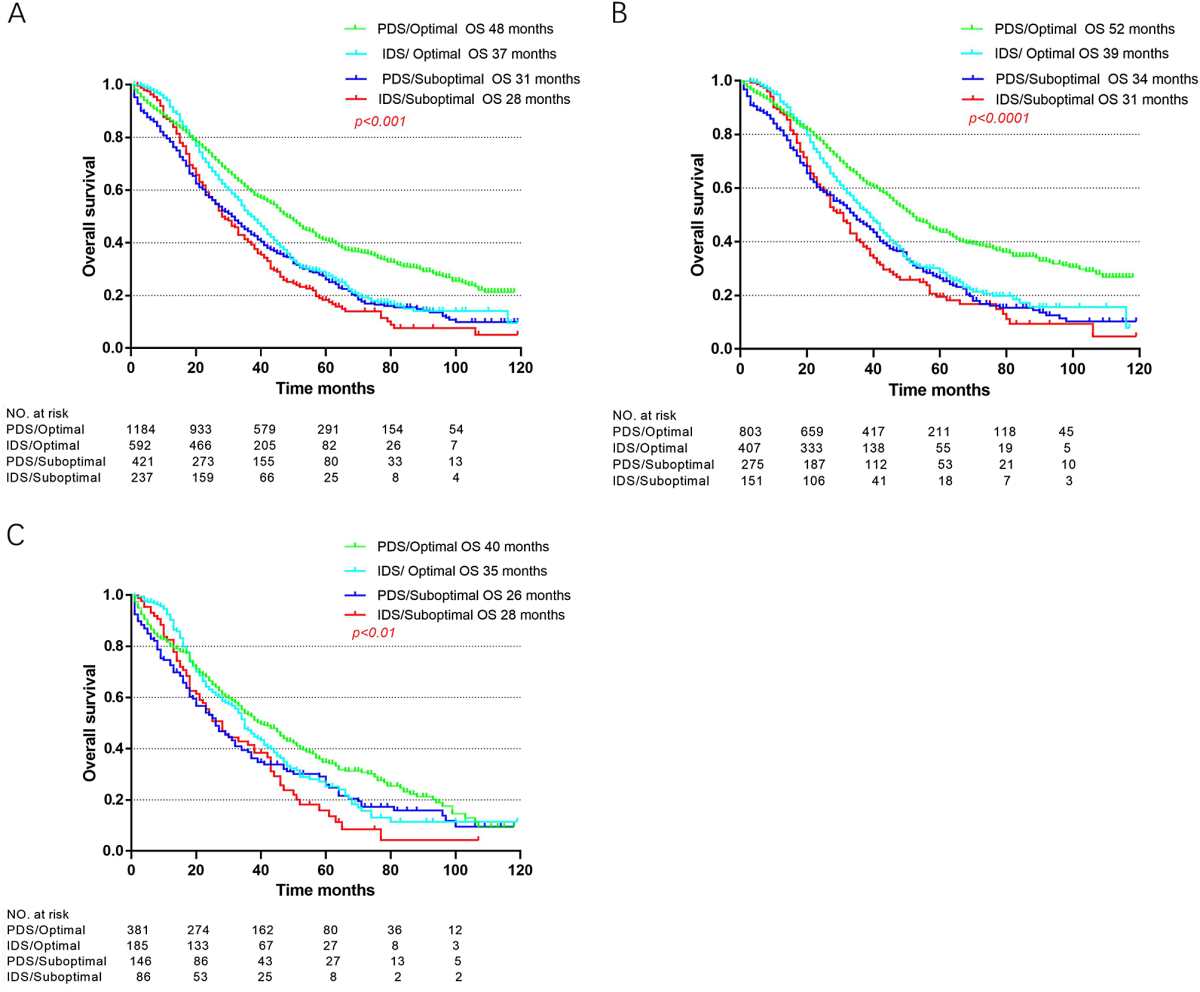
Stratified survival analyses by residual disease. (**A**) Overall survival of all advanced stage patients. (**B**) Overall survival of the elderly group. (**C**) Overall survival of the very elderly group. Abbreviations: PDS, primary debulking surgery and IDS, interval debulking surgery

The associations of treatment types and residual disease status with survival among those with advanced disease were further examined in sub cohorts (Figs. 2 and 3). In terms of treatment type, chemotherapy and surgery were associated with improved OS, which resulted in the best median OS (41 months) (Fig. 2). As shown in Fig. 3, OS was evaluated according to residual disease status. In all advanced-stage patients, PDS with optimal residual disease led to the best survival outcomes. Among patients aged 75 or older, those who underwent IDS had improved survival, with a survival time of 35 months with optimal IDS and 28 months with suboptimal IDS; however, the survival time was 26 months with suboptimal PDS. This trend was not observed among the elderly patients.

**Table 1:** Clinical characteristics of women stratified by age, N (%)

		Age group (Age at diagnosis, years)		*p*-Value
Characteristics	Total (*n* = 5055)	65–74 (*n* = 3190)	≥ 75 (*n* = 1865)	
Marital status				< 0.001
Married Not married Unknown	2488(49.2) 2370(46.9) 197(3.9)	1750(54.9) 1315(41.2) 125(3.9)	738(39.6) 1055(56.5) 72(3.9)	
Race/ethnicity				0.133
White Black Asian or Pacific Islander American Native Unknown	4338(85.8) 322(6.4) 348(6.9) 31(0.6) 16(0.3)	2710(85.0) 218(6.8) 229(7.2) 20(0.6) 13(0.4)	1628(87.3) 104(5.6) 119(6.4) 11(0.6) 3(0.2)	
Laterality				< 0.001
Unilateral Bilateral	2400 (47.5) 2655(52.5)	1443(45.2) 1747(54.8)	957(51.3) 908(48.7)	
Stage				< 0.001
I II III IV Unknown	330(6.5) 455(9.0) 2691(53.2) 1469(29.1) 110(2.2)	222(7.0) 277(8.7) 1702(53.4) 939(29.4) 50(1.6)	108(5.8) 178(9.5) 989(53) 530(28.4) 60(3.2)	
Ca-125				0.324
Negative Positive Unknown	235(4.6) 3996(79.1) 824 (16.3)	150(4.7) 2539(79.6) 501(15.7)	85(4.6) 1457(78.1) 323(17.3)	
Tumor size				0.003
≤50 mm 51-100 mm 101-200 mm >200 mm Unknown	1135(22.5) 1359(26.9) 1096(21.7) 123(2.4) 1342(26.5)	746(23.4) 880(27.6) 699(21.9) 76(2.4) 789(24.7)	389(20.9) 479(25.7) 397(21.3) 47(2.5) 553(29.7)	
Lymph node status				< 0.001
Negative Positive No examined Unknown	1270(25.1) 1027(20.3) 2663(52.7) 95(1.9)	890(27.9) 693(21.7) 1549(48.6) 58(1.8)	380(20.4) 334(17.9) 1114(59.7) 37(2.0)	
Chemotherapy				< 0.001
Yes No	4309(85.2) 746(14.8)	2851(89.4) 339(10.6)	1458(78.2) 407(21.8)	

**Table 2:** Treatment details for women with advanced stage ovarian cancer stratified by age, N (%)

		Age group (Age at diagnosis, years)		*p*-Value
Characteristics	Total (*n* = 3584)	65–74 (*n* = 2296)	≥ 75 (*n* = 1288)	
Stage				0.921
IIIC IV	2127(59.3) 1457(40.7)	1364(59.4) 932(40.6)	763(59.2) 525(40.8)	
LND				< 0.001
Non-LN 1–9 LNs 10–19 LNs ≥ 20 LNs Unknown	1963(54.8) 841(23.5) 378(10.5) 318(8.9) 163(2.3)	1197(52.1) 555(24.2) 255(11.1) 236(10.3) 53(2.3)	766(59.5) 286(22.2) 123(9.5) 82(6.4) 31(2.4)	
Surgery method				< 0.001
No surgery USO/BSO ± Hys USO/ BSO&Ome ± Hys	363(10.1) 290(8.1) 587(16.4)	157(6.8) 172(7.5) 377(16.4)	206(16.0) 118(9.2) 210(16.3)	
Debulking Pelvic exenteration	2250(62.8) 94(2.6)	1519(66.2) 71(3.1)	731(56.8) 23(1.8)	
Treatment type				< 0.001
Chemo + surgery Surgery Chemo No chemo or surgery	2860(79.8) 361(10.1) 261(7.3) 126(2.8)	1951(85.0) 188(8.2) 124(5.4) 33(1.4)	909(70.6) 173(13.4) 137(10.6) 69(5.4)	
NACT				0.212
No Yes	1801(63.0) 1059(37.0)	1244(63.8) 707(36.2)	557(61.3) 352(38.7)	
Residual disease				< 0.001
PDS/Optimal IDS/ Optimal PDS/Suboptimal IDS/Suboptimal No surgery Unknown	1184(33.0) 592(16.5) 421(11.7) 237(6.7) 363(10.1) 787(22.0)	803(35.0) 407(17.7) 275(12.0) 151(6.6) 157(6.8) 503(21.9)	381(29.6) 185(14.4) 146(11.3) 86(6.7) 206(16.0) 284(22.0)	

LNs, lymph nodes; LND, lymphadenectomy; USO, unilateral oophoro-salpingectomy; BSO, bilateral oophoro-salpingectomy; Hys, hysterectomy; Ome, omentectomy; NACT: neoadjuvant-chemotherapy; PDS, primary debulking surgery; IDS, interval debulking surgery.

## Discussion

The present study provides useful insight into the treatment of older women with high-grade serous EOC in the real world and highlights patients older than 75 years of age received significantly fewer standard treatments. Very elderly patients were found to receive less intensive surgery. Cytoreductive surgery rates and optimal cytoreduction rates decrease with increasing age. Similarly, very elderly patients were less likely to receive chemotherapy than younger patients. As a result of the higher FIGO stage and less optimal treatment, very elderly patients with ovarian cancer have a significantly worse prognosis. In our study, 36.2% of the patients aged 65–74 years received NACT, while 38.7% of the patients aged 75 years or older received NACT. For very elderly patients, the survival outcomes were similar between PDS and IDS.

As in prior studies, epithelial ovarian cancer patients with older age have a poorer prognosis [12, 13]. It appears that surgeons tend to perform less radical surgery on older patients with more comorbidities because they consider them frailer. However, for patients with advanced EOC, cytoreductive surgery with no visible tumor has long been considered one of the most important prognostic factors [14]. Bristow et al. established that a 10% increase in maximal cytoreductive surgery is linked to a 5.5% increase in the median survival period [15]. Our study showed the very elderly patients could benefit from complete cytoreductive surgery. Recent literature, however, due to the higher risk of postoperative morbidity and mortality, has discussed the ability and meaning of achieving optimal cytoreduction in older patients [16]. One study showed that the rate of perioperative death within 30 days was 8.2% among patients aged 65 years or older [17]. Additionally, another study found that complication rates increased with age, from 17.1% among women aged 50 to 29.7% among women aged 70–79 and to 31.5% among women aged 80 or older (*P* < 0.05) [18].

High-grade EOC is highly sensitive to chemotherapy. Therefore, even if older patients find radical treatment difficult, chemotherapy is actively selected for curative purposes or symptom relief. Older patients often do not receive standard chemotherapy compared to younger patients because of multiple comorbidities, poor physical or cognitive performance, and the risk of mortality. It is suggested that carboplatin monotherapy or weekly carboplatin in combination with paclitaxel may be considered as a viable alternative to the three-week regimen of carboplatin plus paclitaxel for older patients with ovarian cancer who are susceptible to adverse effects. However, according to the findings of the EWOC-1 trial, carboplatin monotherapy exhibits lower efficacy compared to the weekly or three-week regimens, leading to significantly inferior survival outcomes in susceptible older patients. Consequently, the combination of carboplatin and paclitaxel remains the established standard treatment for older patients [19].

A prior study conducted by Taylor et al [20] showed that among the 9016 patients aged 66 years and older diagnosed with advanced ovarian cancer, the median OS was 10.0 months for those who received chemotherapy alone and 2.0 months for those who underwent surgery. But this trend appears only among the very elderly patients in our study. It may because patients who received chemotherapy alone were more vulnerable than those who could afford surgery, and postoperative mortality has declined in recent years [21].

NACT, which is chemotherapy that is received prior to cytoreduction surgery, is becoming more common in both the United States and Europe, particularly for more vulnerable and older patients, because it is associated with fewer surgical complications. Based on a study of stage II-IV EOC patients, NACT was used by 31.8% of patients in 2007 and 19.7% of patients in 1991 [22]. A study of older patients (66 years or older) diagnosed between 2000 and 2013 with stage III-IV EOC found that the use of NACT increased from 16% in 2000 to 35.4% in 2013, there were no survival differences between NACT and PDS in patients with stage IV disease or for women aged greater than 80 [23]. As IDS after NACT was less invasive than PDS and led to the same survival outcomes among older patients, NACT is recommended for patients over 75 years old by the ASCO and Society of Gynecologic Oncology [24].

A patient’s chronological age is not an adequate surrogate marker, and multidimensional evaluations are essential to fully understand their overall health and tolerance. The Geriatric Assessment (GA) has been recommended by the American Society of Clinical Oncology for all patients who are 65 years old or above and are being considered for chemotherapy. An older adult’s medical, psychosocial, cognitive, and functional capabilities can be evaluated using the GA, which is a multidimensional, multidisciplinary tool. To obtain estimates of chemotherapy toxicity risk, it is advised to use either the Cancer and Aging Research Group (CARG) or Chemotherapy Risk Assessment Scale for High-Age patients (CRASH) tools, while mortality prediction can be aided by the Geriatric-8 or Vulnerable Elders Survey-13 [25].

This study had the advantage of analyzing data from a large sample, allowing for robust statistical analysis. Additionally, to avoid potential confounding effects, the statistical model considered treatment type when assessing the impact of age on OS. A typical histology of high-grade serous EOC was used in our cohort to identify factors associated with poor outcomes in older patients, and further analysis focused mainly on advanced-stage disease. This analysis included patients from SEER’s diverse treatment areas, potentially allowing for broader generalizations than a single-institution investigation on a smaller scale.

Nonetheless, this study has a few limitations. First, EOC management heavily relies on chemotherapy, yet the specifics regarding the regimen type, precision, and adverse effects were not accessible. Second, SEER did not provide more detailed information about tumor markers, molecular testing results, or genetic alterations, which are all important factors in determining a patient’s prognosis. Recent NCCN guidelines have incorporated these tests into treatment algorithms. Third, ovarian cancer has a high recurrence rate, and it is very important to treat it properly after recurrence as well; however, recurrence information was not provided in the SEER database. Finally, we recognize that retrospective studies are subject to selection bias.

There is potential for further improvement in the care of older EOC patients based on the large number of older patients without standard treatment and the large survival gap. In fact, a recent study from the Netherlands looked at older women with advanced stage ovarian cancer and found that, the proportion of older patients receiving oncologic treatment has decreased over the past 12 years [21]. older patients may be at risk for undertreatment. Many randomized clinical trials exclude older patients, despite the fact that ovarian cancer primarily affects the older. Study design and recruitment are more challenging for older patients because they have low performance scores and comorbidities. As a result, evidence-based guidelines often focus on the outcomes of younger patients, while little is known about how to treat older patients optimally. There is a need for more substantial numbers of older patients in clinical rials, as well as prospective designs that specifically target this group of patients.

## Conclusion

The survival of women with EOC strongly decreases with increasing age; noticeably fewer EOC patients aged over 75 years received standard treatment, and more very elderly patients were treated with NACT. To reduce treatment disparities, more knowledge is urgently needed to characterize and identify modifiable factors among older EOC patients.

In a paper conducted by Walree et al. showed most older women did not receive guideline-adherent care and patient preference was the most common reason for this decision [26]. Gynecological oncologists should inform older patients about the increased rates of complications associated with surgery and chemotherapy. Advise older patients on ways to improve their health upon diagnosis and encourage integrated care with geriatrics and palliative care. This approach aims to enhance the quality of life and prioritize the goals of care, considering the guarded prognosis of ovarian cancer in older patients.

### Electronic supplementary material

Below is the link to the electronic supplementary material.

Supplementary Material 1

## Data Availability

The datasets generated and/or analyzed during the current study are available in the Surveillance, Epidemiology, and End Results database, [https://seer.cancer.gov/].

## Abbreviations

EOC: Epithelial ovarian cancer
SEER database: Surveillance, Epidemiology, and End Results database
OS: Overall survival
OCSS: Ovarian cancer-specific survival
CA125: Cancer antigen 125
NACT: Neoadjuvant chemotherapy
PDS: Primary debulking surgery
IDS: Interval debulking surgery
CGR: Complete gross resection

## References

[1] Siegel RL, Miller KD, Wagle NS, Jemal A (2023). Cancer statistics, 2023. CA Cancer J Clin.

[2] Wright JD, Chen L, Tergas AI, Patankar S, Burke WM, Hou JY (2015). Trends in relative survival for ovarian cancer from 1975 to 2011. Obstet Gynecol.

[3] Tew WP (2016). Ovarian cancer in the older woman. J Geriatric Oncol.

[4] Moschetta M, Boussios S, Rassy E, Samartzis EP, Funingana G, Uccello M (2020). Neoadjuvant treatment for newly diagnosed advanced ovarian cancer: where do we stand and where are we going?. Ann Transl Med.

[5] Horowitz NS, Miller A, Rungruang B, Richard SD, Rodriguez N, Bookman MA (2015). Does aggressive surgery improve outcomes? Interaction between preoperative disease burden and complex surgery in patients with advanced-stage ovarian cancer: an analysis of GOG 182. J Clin Oncology: Official J Am Soc Clin Oncol.

[6] Yunokawa M, Onda T, Ishikawa M, Yaegashi N, Kanao H (2022). Current treatment status of older patients with gynecological cancers. Jpn J Clin Oncol.

[7] Nightingale G, Schwartz R, Kachur E, Dixon BN, Cote C, Barlow A (2019). Clinical pharmacology of oncology agents in older adults: a comprehensive review of how chronologic and functional age can influence treatment-related effects. J Geriatric Oncol.

[8] Dumas L, Bowen R, Butler J, Banerjee S. Under-treatment of older patients with newly diagnosed epithelial ovarian Cancer remains an issue. Cancers (Basel). 2021;13. 10.3390/cancers13050952 PMC7956315 33668809

[9] Hurria A, Dale W, Mooney M, Rowland JH, Ballman KV, Cohen HJ (2014). Designing therapeutic clinical trials for older and frail adults with cancer: U13 conference recommendations. J Clin Oncology: Official J Am Soc Clin Oncol.

[10] Prat J (2012). Ovarian carcinomas: five distinct diseases with different origins, genetic alterations, and clinicopathological features. Virchows Archiv: Int J Pathol.

[11] Meinhold-Heerlein I, Fotopoulou C, Harter P, Kurzeder C, Mustea A, Wimberger P (2016). The new WHO classification of ovarian, fallopian tube, and primary peritoneal cancer and its clinical implications. Arch Gynecol Obstet.

[12] Petignat P, Fioretta G, Verkooijen HM, Vlastos AT, Rapiti E, Bouchardy C (2004). Poorer survival of elderly patients with ovarian cancer: a population-based study. Surg Oncol.

[13] Moore KN, Reid MS, Fong DN, Myers TK, Landrum LM, Moxley KM (2008). Ovarian cancer in the octogenarian: does the paradigm of aggressive cytoreductive surgery and chemotherapy still apply?. Gynecol Oncol.

[14] Elattar A, Bryant A, Winter-Roach BA, Hatem M, Naik R (2011). Optimal primary surgical treatment for advanced epithelial ovarian cancer. Cochrane Database Syst Rev.

[15] Bristow RE, Tomacruz RS, Armstrong DK, Trimble EL, Montz FJ (2002). Survival effect of maximal cytoreductive surgery for advanced ovarian carcinoma during the platinum era: a meta-analysis. J Clin Oncology: Official J Am Soc Clin Oncol.

[16] Fanfani F, Fagotti A, Salerno MG, Margariti PA, Gagliardi ML, Gallotta V (2012). Elderly and very elderly advanced ovarian cancer patients: does the age influence the surgical management?. Eur J Surg Oncology: J Eur Soc Surg Oncol Br Association Surg Oncol.

[17] Wright JD, Lewin SN, Deutsch I, Burke WM, Sun X, Neugut AI (2011). Defining the limits of radical cytoreductive surgery for ovarian cancer. Gynecol Oncol.

[18] Bun S, Yunokawa M, Ebata T, Kobayashi Kato M, Shimoi T, Kato T (2019). Feasibility of initial treatment in elderly patients with ovarian cancer in Japan: a retrospective study. Int J Clin Oncol.

[19] Falandry C, Rousseau F, Mouret-Reynier MA, Tinquaut F, Lorusso D, Herrstedt J (2021). Efficacy and safety of first-line single-Agent Carboplatin vs Carboplatin Plus Paclitaxel for Vulnerable Older Adult Women with Ovarian Cancer: a GINECO/GCIG randomized clinical trial. JAMA Oncol.

[20] Taylor JS, He W, Harrison R, Zhao H, Sun CC, Lu KH (2018). Disparities in treatment and survival among elderly ovarian cancer patients. Gynecol Oncol.

[21] Schuurman MS, Kruitwagen R, Portielje JEA, Roes EM, Lemmens V, van der Aa MA (2018). Treatment and outcome of elderly patients with advanced stage ovarian cancer: a nationwide analysis. Gynecol Oncol.

[22] Wright JD, Ananth CV, Tsui J, Glied SA, Burke WM, Lu YS (2014). Comparative effectiveness of upfront treatment strategies in elderly women with ovarian cancer. Cancer.

[23] Meyer LA, He W, Sun CC, Zhao H, Wright AA, Suidan RS (2018). Neoadjuvant chemotherapy in elderly women with ovarian cancer: rates of use and effectiveness. Gynecol Oncol.

[24] Wright AA, Bohlke K, Armstrong DK, Bookman MA, Cliby WA, Coleman RL (2016). Neoadjuvant chemotherapy for newly diagnosed, advanced ovarian cancer: Society of Gynecologic Oncology and American Society of Clinical Oncology Clinical Practice Guideline. Gynecol Oncol.

[25] Mohile SG, Dale W, Somerfield MR, Schonberg MA, Boyd CM, Burhenn PS (2018). Practical Assessment and Management of vulnerabilities in older patients receiving chemotherapy: ASCO Guideline for Geriatric Oncology. J Clin Oncology: Official J Am Soc Clin Oncol.

[26] van Walree IC, Bretveld R, van Huis-Tanja LH, Louwers JA, Emmelot-Vonk MH, Hamaker ME (2020). Reasons for guideline non-adherence in older and younger women with advanced stage ovarian cancer. Gynecol Oncol.

