# Supplementary material for: Disparities in treatment modalities and survival among older patients with high-grade serous ovarian cancer
Source: BMC Womens Health. 2024 Feb 7;24:100. doi: 10.1186/s12905-024-02938-y (PMC10851467; doi:10.1186/s12905-024-02938-y)
Supplement: Supplementary file 1 — Supplementary Material 1 [file 12905_2024_2938_MOESM1_ESM.pdf]

Supplementary tables and figures

Table 1. Cohort selection

Selection Criteria	Included / Remaining	Excluded
Ovarian cancer （Year of diagnosis 2010-2017）	44389	
Prior cancer diagnosis	37706	6683
Age of diagnosis, ≥20years	37128	578
Available follow-up time and Survival months≥1	33242	3886
Microscopically confirmed	32083	1159
Serous or Endometrioid ovarian cancer (known grade)	15242	16841
Grade 3 and 4	11626	3616
Age of diagnosis, ≥65years	5055	6571
**Final Cohort**	5055	

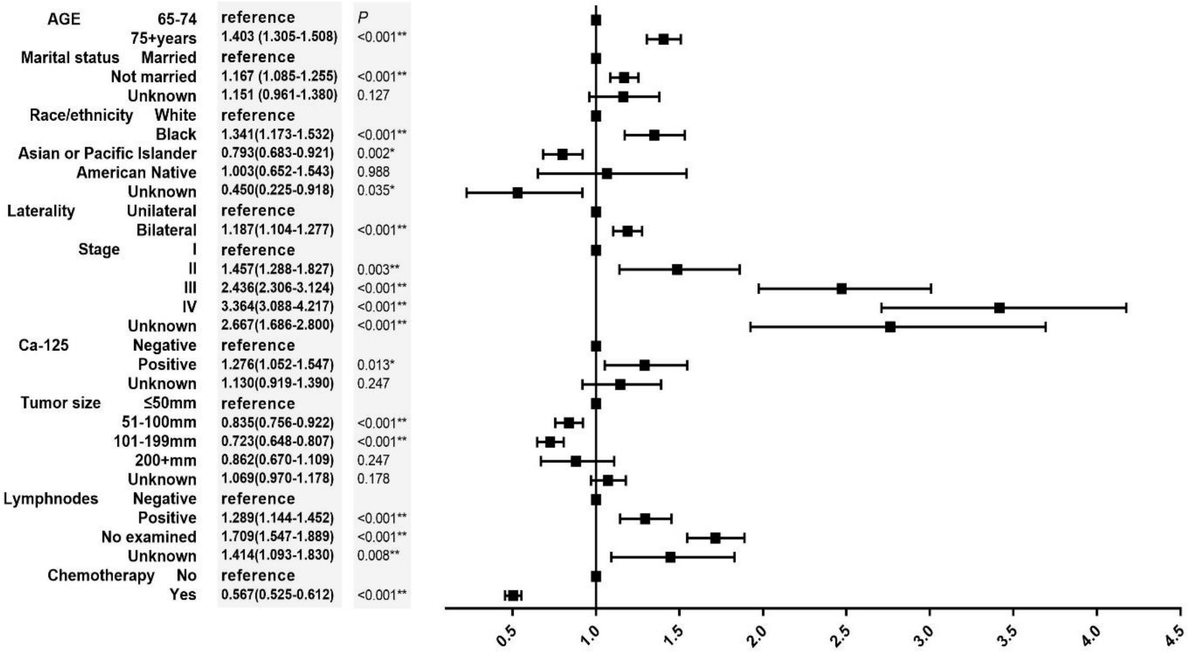

Figure 1. Multivariate survival analysis of all patients.

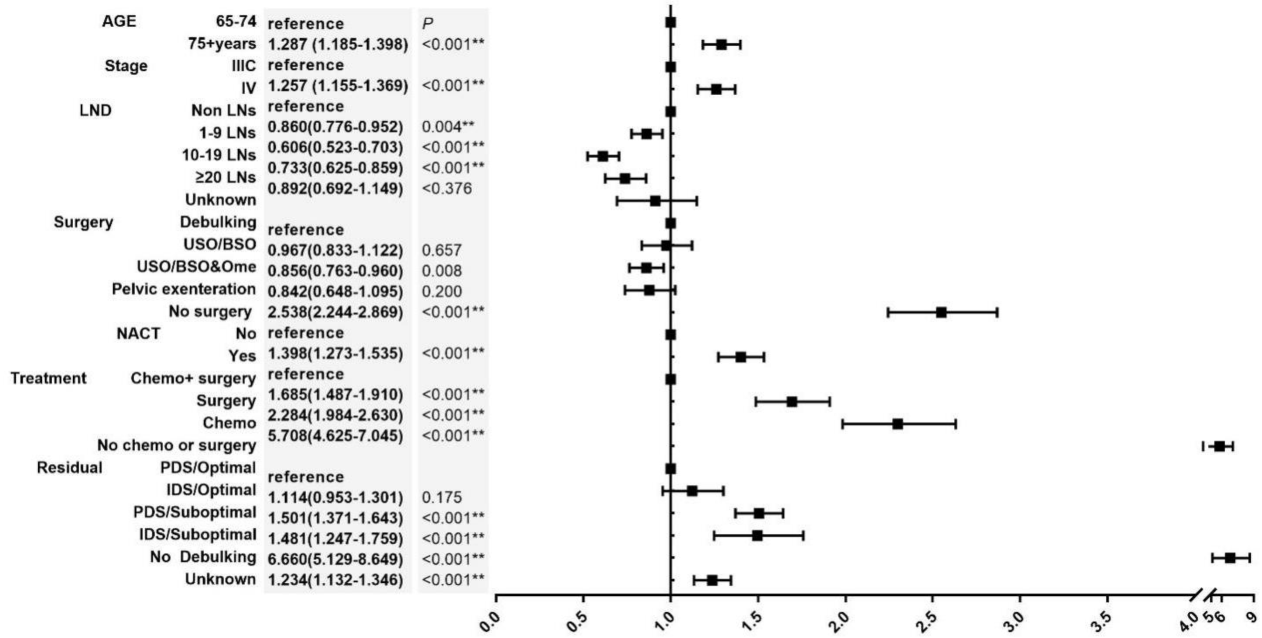

Figure 2. Multivariate survival analysis of advanced stage patients.
